# Double Skull Sign After Cranioplasty: A Case Report

**DOI:** 10.7759/cureus.57892

**Published:** 2024-04-09

**Authors:** Hina Hamada, Koji Hayashi, Asuka Suzuki, Yuka Nakaya, Toyoaki Miura, Mamiko Sato, Yasutaka Kobayashi

**Affiliations:** 1 Department of Rehabilitation Medicine, Fukui General Hospital, Fukui, JPN; 2 Graduate School of Health Science, Fukui Health Science University, Fukui, JPN

**Keywords:** diagnostic ct imaging, skull, artificial dura, epidural fluid collection, double skull sign, cranioplasty

## Abstract

The double skull sign (DSS) is a unique image on the outside of the brain that looks like two skulls. Whereas congenital and acquired types of DSS have been reported, the etiology of both of them is calcified hematomas. We encountered a case of a 46-year-old woman with a history of subarachnoid hemorrhage followed by cranioplasty at 43 years old. She developed right hemiparalysis and motor aphasia suddenly. Brain computed tomography and magnetic resonance imaging revealed not only cerebral infarction but also DSS incidentally. After detailed analysis, we concluded that the DSS in this case was not due to calcification of the hematoma but was related to the cranioplasty. In this report, we present an interesting case and discuss etiologies of the development of DSS after cranioplasty.

## Introduction

The double skull sign (DSS) is a unique image on brain computed tomography (CT) that shows an eggshell-like calcification and two skulls on the outside of the brain [1]. Although the abnormity of the skull is generally caused by several reasons, such as impairment in embryological process or development, acquired diseases, post-traumatic craniofacial abnormalities, neoplasia, deformations, or sutural synostosis [2], it has been reported that DSS is related to the calcified hematomas [1,3,4]. Congenital and acquired types of DSS have been reported, and both of them are caused by hematomas, including cephalohematomas, chronic subdural hematomas, or extradural hematomas [3,4]. The congenital type exhibited a deformed skull, particularly with an outward convex-like malformation [3]. The reason for this outward convex-like skull deformity in the congenital type is believed to be the softness of the baby's skull, which causes the skull malformation in addition to the cephalohematoma during vacuum delivery [3]. On the other hand, there is no skull deformity in the acquired type, because acquired DSS is caused by ossified chronic subdural hematomas or extradural hematomas after a hard skull is completed [3]. In this report, we present an interesting case with DSS caused not by hematomas but post cranioplasty.

## Case presentation

**Figure 1:**
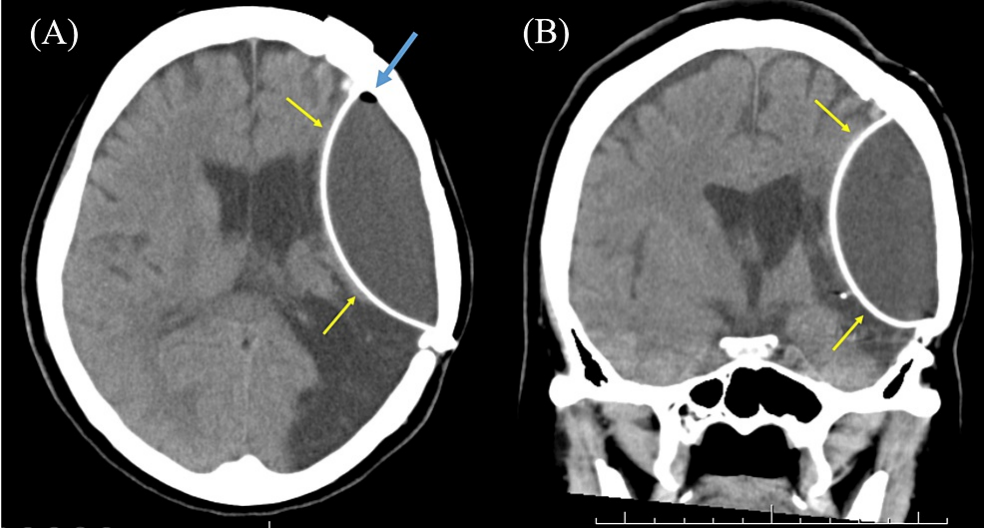
The result of brain computed tomography (CT). Brain CT showing a double skull sign (DSS) in the right hemisphere (yellow arrowheads). In addition, a small amount of air is noted inside the DSS (blue arrowhead). (A) Axial section; (B) coronal section.

**Figure 2:**
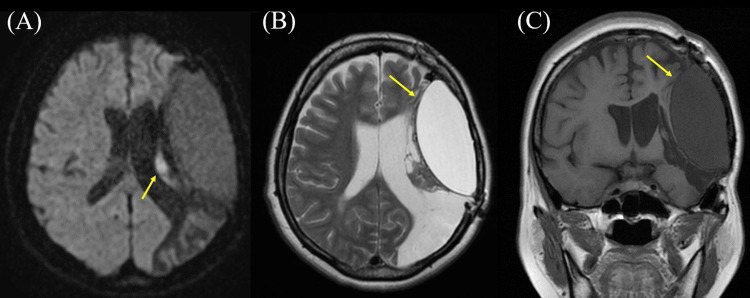
The result of brain magnetic resonance imaging (MRI). (A) Diffusion-weighted brain MRI on admission showing hyperintensity in the left corona radiata near the double skull sign (DSS) (arrowhead). (B) T2-weighted brain MRI (axial section) 2.5 months after admission showing hyperintensity inside DSS (arrowhead). (C) T1-weighted brain MRI (coronal section) 2.5 months after admission showing hypointensity inside DSS (arrowhead).

A 46-year-old woman developed right hemiparalysis and motor aphasia suddenly. She had a history of subarachnoid hemorrhage (SAH) at 43 years old and was treated with cranioplasty at another hospital. However, we could not obtain detailed information on cranioplasty, including materials and surgical method used. Although she had slight hemiparalysis in the right body and mild motor aphasia as residual symptoms after SAH, she returned to work, managed the company, and regained independence in her activities of daily living. She was transported to the previous hospital due to exacerbated symptoms triggered by a new episode. Brain CT revealed a DSS in the left hemicerebrum (Figure 1). In addition, a small amount of air was observed in the content of DSS (Figure 1A). Brain MRI revealed hyperintensity in the left corona radiata on diffusion-weighted imaging (Figure 2A), and the DSS content manifested as hyperintensity on T1-weighted images and hyperintensity on T2-weighted images (Figures 2B, 2C).

She was treated with antiplatelets (ozagrel sodium: 160 mg/day, followed by cilostazol 200 mg/day) as acute medication treatment. She was transferred to our hospital for rehabilitation therapy on day 37. She was treated with rehabilitation therapy for about five months. She was discharged from our hospital and had recovered sufficiently to carry out daily activities, although mild hemiparesis persisted.

## Discussion

This report presents a case of DSS on brain CT and MRI. The patient had a history of cranioplasty for SAH three years before admission. Although she was admitted to our hospital for cerebral infarction, DSS was incidentally identified. On brain CT and MRI, the DSS contents were considered to be homogeneous liquid, with some air present. In addition, MRI findings revealed that the internal contents of DSS showed hyperintensity by T2-weighted images and hypointensity by T1-weighted images, which seemed to be not a hematoma, but a water-like component. Another skull (pseudo-skull) that existed inside the true skull had a smooth surface. It seemed that the patient had DSS prior to this hospitalization, but it was unclear whether DSS was causing any symptoms.

After cranioplasty, major complications include infections, seizures, bone flap resorption, intra-cranial hemorrhages, and epidural fluid collections (EFC) noted in the early postoperative period [5]. The incidence of EFC after cranioplasty is relatively high, estimated at 37.3-79.2% [5]. Whereas the etiology of EFC following cranioplasty remains unclear, a few reports speculate its mechanisms including allergic reaction for allograft materials [6], foreign body reaction [7], cerebrospinal fluid leak [8], and an air bubble in the epidural space initiating an inflammatory process, resulting in the formation of exudates [8,9].

In our case, the contents of the DSS appeared to be fluid rather than hematoma, based on the brain images. Additionally, a small amount of air was observed within the DSS. Moreover, it appears that the pseudo-skull is composed of artificial materials rather than calcification, as the surface is smooth. On the basis of previous reports [5-9], we hypothesized three possible mechanisms of developing DSS after cranioplasty. First is the relation with air bubbles. The air bubbles that existed between the artificial dura and the real skull might have triggered EFC, causing the artificial dura to separate. Indeed, artificial dura mater shows high density and smooth surface on CT [10]. Therefore, it is possible that pseudo-skull and DSS were noted on brain images, including CT and MRI. Second is the shift of artificial dura mater, namely, pseudo-skull, toward the brain. The artificial dura mater, which was attached to the real skull, might come off and move inward, resulting in the formation of a pseudo-skull [5]. Although the detailed mechanism remains unknown, fluid collection might be observed in this empty space. This fluid retention may be caused by fluid building up to fill an empty space or by an allergic or foreign body reaction to the cranioplasty material [6,7]. Third, the contents may have once been a hematoma. One of the complications of cranioplasty is hemorrhage [5], and hematoma might have formed between the true skull and the pseudo-skull. MRI signals of hematomas change over time, and the signals gradually become very hyperintense like CSF [11]. Although the third mechanism is quite conceivable, there was no hypointensity area on the brain T2-weighted MRI inside DSS in our case (images are not shown).

This report has two major limitations. Firstly, we cannot analyze the contents of DSS. Therefore, we cannot exclude the possibility of a CSF leak in relation to DSS development. If we had been able to test for the presence of sugar, we would have been able to distinguish whether the contents were the same as CSF. Alternatively, checking the contents of the DSS might have provided evidence of a hematoma. Secondly, we were unable to obtain the operative record of the cranioplasty. The cranioplasty was performed at another hospital, and although we made inquiries regarding the procedure in detail, we could not obtain an answer as the surgeon had been transferred. If the material of the artificial dura mater were susceptible to allergic reactions, it would be easier to speculate about the mechanism of DSS occurrence.

To date, there are no papers reporting DSS after cranioplasty. Although the appearance on the brain images was similar to previously reported DSS caused by calcified hematoma, we speculated that the artificial dura mater appeared separated from the true skull in the present case. Therefore, this case might not be classified into DSS in terms of classical concepts. However, clinicians may encounter similar cases with the appearance of DSS and may need clues to investigate its etiology. We believe that this report will provide insights for clinicians to understand the mechanisms of developing DSS. In addition, this report may extend the comprehension of DSS.

## Conclusions

We presented a case with DSS after cranioplasty. When the artificial dura separates from the true skull, it may look like a pseudo-skull. Although detailed mechanisms remain unknown, as one of the potential mechanisms, air bubbles might induce EFC between the true skull and pseudo-skull, resulting in the manifestation of DSS on brain images. Although previous reports only mentioned DSS in relation to hematomas, this report may broaden the understanding of DSS. We suggest that cranioplasty be newly added as an etiology to the list of causes of DSS.
